# Transmissibility of the Influenza Virus in the 1918 Pandemic

**DOI:** 10.1371/journal.pone.0001498

**Published:** 2008-01-30

**Authors:** Laura Forsberg White, Marcello Pagano

**Affiliations:** Department of Biostatistics, Boston University School of Public Health, Boston, Massachusetts, United States of America; University of Liverpool, United Kingdom

## Abstract

**Background:**

With a heightened increase in concern for an influenza pandemic we sought to better understand the 1918 Influenza pandemic, the most devastating epidemic of the previous century.

**Methodology/Principal Findings:**

We use data from several communities in Maryland, USA as well as two ships that experienced well-documented outbreaks of influenza in 1918. Using a likelihood-based method and a nonparametric method, we estimate the serial interval and reproductive number throughout the course of each outbreak. This analysis shows the basic reproductive number to be slightly lower in the Maryland communities (between 1.34 and 3.21) than for the enclosed populations on the ships (R_0_ = 4.97, SE = 3.31). Additionally the effective reproductive number declined to sub epidemic levels more quickly on the ships (within around 10 days) than in the communities (within 30–40 days). The mean serial interval for the ships was consistent (3.33, SE = 5.96 and 3.81, SE = 3.69), while the serial intervals in the communities varied substantially (between 2.83, SE = 0.53 and 8.28, SE = 951.95).

**Conclusions/Significance:**

These results illustrate the importance of considering the population dynamics when making statements about the epidemiological parameters of Influenza. The methods that we employ for estimation of the reproductive numbers and the serial interval can be easily replicated in other populations and with other diseases.

## Introduction

The emergence of the highly pathogenic avian influenza strain H5N1 has raised concerns of an imminent influenza pandemic. Public health workers, government officials and disaster planners have an increasing interest in better understanding the potential impact of an influenza pandemic and possible strategies for containment. Crucial in this planning is an understanding of the basic epidemiology of the disease in various settings. This has led to a growing interest in the analysis and understanding of past epidemics, particularly that of 1918, the most virulent and deadly influenza epidemic of the 20th century. Mortality has been estimated at 50–100 million people worldwide as a result of influenza in the 1918 pandemic [1]. It is reasonable to suppose that by better understanding the transmission dynamics of the highly pathogenic virus in 1918, we can gain greater insight into the dynamics, and thus potential methods of control, for a future pandemic [2].

Important parameters for understanding disease transmission are the reproductive number and the serial interval [3]. The basic reproductive number is defined as the average number of secondary infections created from a primary infection in an entirely susceptible population [4], [see also 5]. A more complex, but perhaps meaningful parameter is the effective reproductive number which defines the average number of secondary infections an infected will create at a given point in the epidemic. This parameter takes into account that not all contacts of an infected individual are with susceptible persons, as well as the impact of public health control measures. Control strategies are typically targeted to drive this number below one and maintain it there, as this will lead to eventual extinction of the epidemic. An example of this is herd immunity, or immunity to a disease that is incurred from a sufficiently large proportion of the population being immune to a disease. Modeling techniques are often used to determine the proportion of the population that should be vaccinated in order to have the reproductive number low enough to avoid outbreaks of disease [6].

The serial interval can be defined as the time interval between a primary case presenting with symptoms and its infectee developing symptoms [7], [8]. Thus this quantity is completely observable. This is a mixture of the incubation period and the infectious period, both of which are useful to understand, but difficult to measure. The SARS outbreak of 2003 had a relatively long serial interval, estimated to be between 8 and 10 days on average and following a Weibull distribution [9] making case isolation extremely effective in containing the epidemic.

Methods for the estimation of basic epidemiological parameters are still in development phase. [10] provides a thoughtful summary of methods for estimating the reproductive number. One particularly interesting and useful method has been previously described by [7] for estimating the daily reproductive number, R_t_, or the average number of cases an infected individual on day t would cause. One interesting feature of this method is that for days where no cases are observed, the estimated effective reproductive number is zero. Another observation is that this method essentially estimates a curve for the effective reproductive number that traces the epidemic curve, lagged by the average serial interval length. This nonparametric method presupposes information on the serial interval distribution. This is typical as most methods for estimating the reproductive number rely on knowledge of the serial interval.

Few have described analytical methods for estimating the serial interval, making most methodologies dependent on contact tracing data, which is often difficult and expensive to attain. [11] describe a method to estimate the reproductive number that relies on limited contact tracing information but not a full estimate of the serial interval. [12] have recently described a method to estimate the serial interval and then used this estimate with the estimator proposed in [7] of the daily reproductive number and have applied their method to data from outbreaks of avian influenza in poultry farms in Europe.

Several researchers have studied the 1918 pandemic and estimated some of these key epidemiological parameters. Estimates have ranged from 2–3 for the basic reproductive number, R_0_, when using an SEIR model with a mean latent period of 1.9 days and infectious period of 4.1 days [13], [14]. Using an exponential model and assuming the serial interval to be four days (somewhat based on the assumptions of [13]), [15] estimated R_0 _to be 2.6–10.6 for confined settings (such as prison and ships) and 2.4–4.3 for community settings. The estimates for the mean latent and infectious periods come from [16] and were used again by [17] and [18]. It appears that the original estimates were derived from epidemic data, although their source is not well documented.

In what follows, we introduce new methodology for the estimation of both the daily reproductive number and the serial interval. We apply this method to data from two outbreaks on military ships in the 1918 influenza outbreak, as well as well-documented outbreaks in five Maryland communities. The results from this method are compared to that of [12]. The results illustrate the differences in infectious disease dynamics between outbreaks in a closed population and a dynamic community.

## Methods

### Data

We analyze data from several well-documented influenza outbreaks in 1918. First we consider data from two troop ships that embarked in the late fall of 1918 [19]. The Medic reported two initial cases on November 11. Out of 989 passengers (156 crew members, 829 soldiers, 4 civilians) 313 became sick with influenza over a 40 day period (Attack Rate, AR, = 0.32), though most of the cases occurred within the first fourteen days. The Boonah left Durban and in five days, on November 29, reported the first three definitive cases of influenza. Those who collected the data note that there were likely some initial cases that were not identified. Out of 1095 on board (164 crew members and 931 troops), 470 cases were reported (AR = 0.43) in the 40 days of the epidemic.

The United States Public Health Service created special surveys of 18 localities during the pandemic [20]. Reported results from six communities in Maryland are derived from house-to-house surveys requesting the date of onset of influenza for all infected, and the sex and age of each case of pneumonia and influenza. A summary of these populations is provided in Table 1.

**Table 1:** Demographic and survey information on the Maryland communities surveyed in the 1918 Influenza pandemic.

Community	1917 Population	Number Surveyed	Percent Surveyed	Number of Cases	Attack Rate
Baltimore	594,637	33,776	5.7	7,868	0.23
Cumberland	26,686	5,234	19.6	2,147	0.41
Frederick	11,225	2,420	21.6	777	0.32
Salisbury	6,690*	1,735	25.9*	796	0.46
Lonaconing	1,553*	1,840	-	1,093	0.59

### Statistical Methods

We describe a likelihood based methodology for estimating the reproductive number at each day in the epidemic as well as the serial interval. The method builds on that described by [21]. We assume that the population is closed, that all cases are observed, and use daily case counts only (i.e. number of new cases each day).

Let **N** = {N_0_, N_1_, N_2_,…, N_T_} represent the daily cases counts of influenza for the T days of the epidemic and X_ij_ represent the number of cases that appear on day j that are infected by individuals that appeared sick on day i. Following is a representation of the disease transmission model in the population.
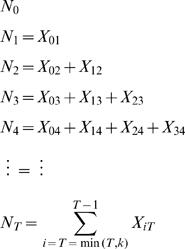



We assume that the total number of cases produced by those on day i, X_i**·**_, are Poisson distributed with parameter N_i_ R_i_, where R_i _is the reproductive number for cases on day i. We further assume that **X**_i_ = {X_i,i+1_, X_i,i+2_,…,X_i,i+k_} follows a multinomial distribution with parameters X_i**·**_, **p**, k, where **p** = {p_1, _p_2,…,_p_k_} represent the distribution of the serial interval. Using these assumptions we can construct a likelihood function (see details in the Supplemental Information), which, when simplified, yields the following convenient form
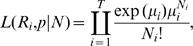
where 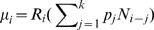 [21].

Maximization of this likelihood with respect to R_i_ and **p** yields estimates of these parameters. To further simplify this process and create a more parsimonious model, we parameterize **p** by allowing it to follow a traditional parametric form for a serial interval (for instance a Weibull, Gamma, Log Normal, or Exponential distribution). Then the p_j_ are functions of the parameters of the density (for instance in the case of the Gamma distribution, the p_j_ only depend on the shape and rate parameters of the Gamma).

Similarly R_i_ can be modeled parametrically as a function of time. One example of a reasonable model for this is the four parameter logistic curve [22]–[24] given by
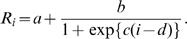



The parameters of this curve describe the initial height of the curve (approximately a+b), the point of inflection (d), the curvature over the inflection (c) and the final height of the curve (a). These parameters have biological meaning in this setting where the initial height corresponds to the values of R_i_ prior to intervention and significant depletion of the susceptible population. The inflection point and its steepness would describe the timing of intervention and the rapidity with which it impacts transmission. The final height would describe the ultimate value of R_i,_ which typically is less than one, indicating that disease transmission is in a sub epidemic state.

In our analysis, we also implement the method described by [12] (hereafter referred to as the Garske et al. method) and compare the results of the two methodologies. This method first estimates the generation time distribution using a likelihood based method. Then the effective reproductive number is estimated using the method described by [7] (hereafter referred to as the WT method). We fit the likelihood for both methods using a Nelder-Mead maximization procedure and use 576 starting values in order to ensure that we reach the global maximum. All analyses were done using R 2.4.1.

Both methods assume homogenous mixing in the population, no missing data (clearly violated with the data from the Maryland communities), that a primary case experiences symptom onset prior to any cases that it infects and a completely closed system where all cases are infected by a case that has been observed. In the case of the Maryland data, where only a sample of the total number of cases was surveyed, we can observe the efficacy and robustness of these methods with sample data. Certainly results should be interpreted with caution, however, as we will show, the results that are obtained are consistent with previous estimates for influenza.

### Error Estimates and Residuals

Standard errors were calculated for the MLE method using a parametric bootstrap. One thousand epidemics were simulated using the parameter estimates and estimates were obtained from each of these simulated epidemics. The standard deviation of the 1000 estimates was used as the standard error estimates. We used the method described in [12] to estimate the standard error for their estimates, however our simulations based on their assumption of asymptotic normality yielded a large number of negative estimates for the parameters. It is possible that this is due to the non-independence in the data and lack of theoretical underpinnings for the method that they propose. These results make their standard error estimates infeasible to estimate in this case. Therefore we do not present standard error estimates for the results obtained using their methodology.

In order to determine the accuracy and relative merit of the estimates obtained from each methodology, we compute one-step-ahead residuals and implement a cross validation approach to analyze the generalizeability of the estimates obtained. The one-step-ahead residuals were calculated by first using the estimates from a particular location along with the data to predict the next days' number of cases, 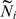 as follows:
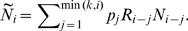



Each 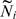 is calculated using N_0_, N_1_, …, N_i−1_. Then the one-step-ahead residuals are calculated as
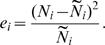
We present these residuals averaged over the T days observed.

Generalizeability of the results was studied using an ad hoc cross validation (CV) technique. This is done by using the estimates obtained from one location to calculate the one step ahead residuals for another location. Specifically we use the Boonah ship estimates to calculate residuals with the Medic data and then use the Medic estimates to calculate the residuals for the Boonah data. For the Maryland communities, we report the average of the residuals obtained using the estimates from one community to predict the epidemics in each of the other four communities, creating five CV estimates (one for each community).

## Results

### Serial Interval Estimates

Table 2 gives the results for the serial interval distribution estimates. Notable in these results is the striking consistency in the estimates of the first moment, with the exception of Cumberland. The second moments vary much more, however. In general they tend to be much larger for the ships when using the Garske et al. method compared to the MLE method. For the communities, we observe that they are consistently around 10 for the Garske et al. method and vary much more for the MLE method. Also of interest in these results are the large error estimates, particularly for Cumberland, but also to a smaller extent for Frederick. This is perhaps indicative of the model not fitting the data as well, for instance the logistic model may not be the best fit in this scenario, or that the lack of census data on cases might be more problematic here.

**Table 2:** Serial interval estimates for the MLE method and the Garske et al. method.

Location	MLE		Garske et al	
	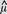 (SE)	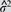 (SE)	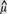	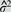
*Ships*				
Boonah	3.81 (3.69)	1.25 (2.83)	4.38	19.64
Medic	3.33 (5.96)	11.35 (4.60)	3.88	18.04
*Communities*				
Baltimore	2.83 (0.53)	2.30 (1.28)	2.90	8.45
Cumberland	8.28 (951.95)	25.00 (6143.28)	3.61	10.71
Frederick	4.66 (10.68)	28.65 (157.78)	3.09	11.57
Salisbury	3.31 (1.94)	9.08 (14.80)	3.76	12.40
Lonaconing	4.02 (14.10)	3.25 (26.74)	3.99	12.69

### Reproduction Number

**Figure 1:**
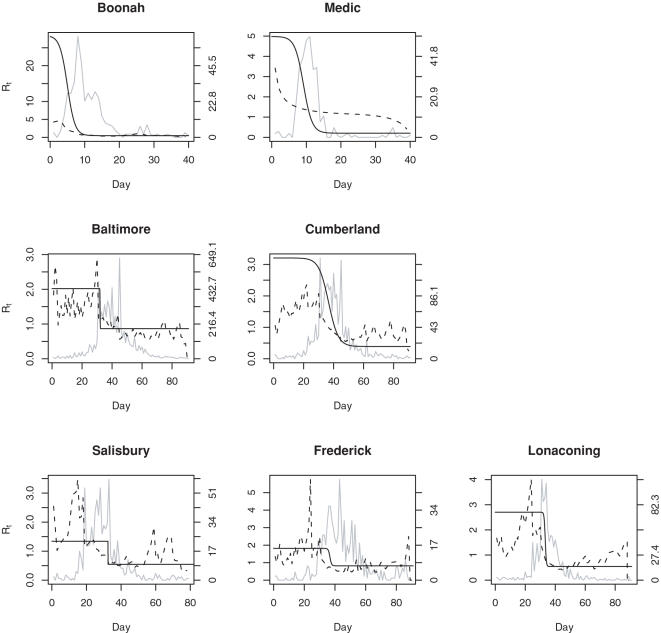
Estimated effective reproductive number for each location, using the MLE method (solid black line) and WT method (dashed line). The epidemic curve is shown in gray and its axis is on the right of the figure.

In Table 3 and Figure 1, we present the results for estimation of the effective reproductive number. Evident in these results, is the large initial reproductive number for the Boonah ship. This is likely due to some of the missing data at the beginning of the epidemic and thus the model attributing the large number of cases that rapidly develop to the few individuals who were initially reported. The logistic model fits this as accurately as possible, but perhaps the important message is the qualitative result, indicating that initial transmission in this susceptible, non-quarantined population was very high and rapidly decreased as many became infected. The result is similar for Medic though the initial value is not high. We also note that the reproductive number dropped to sub epidemic levels rapidly (around 10 days for both ships).

**Table 3:** Effective reproduction number estimates for the MLE method and the Wallinga and Tuenis (WT) method.

Location	Max WT 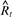 (day)	Day 1	Day 10	Day 30	Day 70
**Ships**					
*Boonah*					
MLE (SE)		27.71 (8.04)	0.74 (0.71)	0.47 (0.03)	
WT	4.74 (3)	4.27	0.68	0.53	
*Medic*					
MLE (SE)		4.98 (3.31)	1.83 (0.830)	0.21 (0.09)	
WT	3.42 (1)	3.42	1.36	1.06	
**Communities**					
*Baltimore*					
MLE (SE)		2.02 (0.12)	2.02 ( 0.12)	2.02 (0.12)	0.87 (0.12)
WT	2.90 (30)				
*Cumberland*					
MLE (SE)		3.21 (0.73)	3.21 (0.73)	2.88 (0.66)	0.39 (0.15)
WT	2.35 (22)	1.06	1.42	2.07	0.84
*Frederick*					
MLE (SE)		1.82 (0.14)	1.82 (0.14)	1.82 (0.14)	0.82 (0.04)
WT	5.76 (24)	1.35	1.11	2.49	1.02
*Salisbury*					
MLE (SE)		1.34 (0.18)	1.34 (0.18	1.34 (0.18)	0.55 (0.13)
WT	3.47 (15)	2.55	1.79	0.86	0.87
*Lonaconing*					
MLE (SE)		2.70 (0.19)	2.70 (0.19)	2.70 (0.19)	0.54 (0.03)
WT	4.01 (24)	1.67	1.17	2.10	0.81

In the Maryland communities the initial reproductive number tended to be slightly lower (ranging from 1.34 in Salisbury to 3.21 in Cumberland). For the WT method, the initial values were also small, but if one considers the maximal values, these were much more varied (from 2.35 to 5.76), as shown in Table 2. In fact, we observed that the effective reproductive number peaked relatively late in the epidemic and assumed much higher values than that observed with the MLE method. Overall it took longer for the reproductive number to drop below one in these communities (typically between 30–40 days).

### Generalizeability

In Table 4, we present the results of the residual analysis. We notice here that the Garske et al. method often does better than the MLE method. It is important to point out that the WT method of fitting the effective reproductive model over fits the model and suffers from generalizeability. This method essentially traces the epidemic curve, lagged by the mean of the generation time distribution. Thus, according to the residuals, it appears that the WT method outperforms the MLE. However, considering the importance of external validation and reproducibility, the model suffers somewhat as evidenced by the CV measures. The exceptions to this are in the case of the Boonah where the CV measure is impacted by the large initial MLE estimate of the reproductive number and in Cumberland where it appears that either the parametric model chosen may not represent the best fit to the data or there were sensitivities to the survey data.

**Table 4:** One step ahead residuals for both methods fitting each epidemic.

Location	Residual		CV Measure	
	MLE	Garske et al	MLE	Garske et al
*Ships*				
Boonah	25.04	3.84	56.57	19.03
Medic	13.27	6.31	15.09	30.30
*Communities*				
Baltimore	29.36	20.54	5.81	5.95
Cumberland	10.01	5.50	18.37	13.23
Frederick	3.80	14.67	13.48	16.37
Salisbury	6.32	3.38	20.08	66.84
Lonaconing	8.21	4.51	16.69	19.14

The cross validation measure for the ships is the one step ahead residuals calculated from predicting one ship's data using the estimates from the other ship's data. For the communities, it is the sum of the residuals predicting the other four communities using the estimates from the community indicated. For instance the CV measure for Baltimore is the sum of all the residuals that come from using the estimates for Baltimore to predict the other four communities' outbreaks.

## Discussion

We have presented results that are informative with regard to the dynamics of the 1918 influenza pandemic in different populations and provide insight into two methodologies for estimating basic epidemiological parameters. Both methods assume that the population is closed, there are no missing cases and no migration to or from the population. The second of these assumptions is clearly violated with the data from Maryland; however the results appear to be reasonably robust to this discrepancy, except in the case of Cumberland.

The purpose of this exercise determines to some extent which methodological approach we might favor. If the intent is to simply estimate the parameters for a specific epidemic and better understand what exactly was occurring in that setting, then the method presented by [12] (Garske et al.) appears to provide good fit. The caveat that we see in this method is that by estimating the effective reproductive number with the methodology of [7] (WT) there is an over fit of this parameter and it essentially traces the epidemic curve, lagged by the mean of the serial interval. It is not clear if this is a desirable or informative property. The MLE method has greater promise for generalizeability. While it can be argued that adhering to a parametric definition of the shape of the effective reproductive number leads to a greater chance of lack of fit, it can also lead to a result that can be interpretable for other settings that are similar to that being studied.

One can choose any reasonable parametric form for modeling the effective reproductive number. Here we have only shown the four parameter logistic model, and feel that it is suitable in most cases where the epidemic curve has a single peak. It is feasible that this model may not apply well in all situations. Another approach might be to analyze the data using the Garske et al. method and then smooth the plot of the effective reproductive number and from this determine a parametric form that closely approximates the smoothed curve. Multiple models could be implemented, then the residual analysis that we have shown provides a valuable tool for model assessment and comparison.

The results of these models can be sensitive to underreporting initially in the epidemic. We see this clearly in Boonah, where it was acknowledged that there was underreporting early on and this led to us getting very high estimates for the initial reproductive number. Similarly, in Cumberland, if we remove the first five days of data (three cases on the first day, six cases on the second and then no cases the following three days) we get much more reasonable estimates (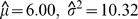) with smaller residuals (6.00). Therefore, it is important to note that unusual observations in the first few days can impact the estimates and one should pay careful attention to this possibility.

Overall both methodologies presented are valuable tools that can be used in tandem for understanding the dynamics of infectious disease epidemics. These methods are easy to implement and interpret.

The results that we have presented suggest that the average serial interval for pandemic influenza in 1918 was consistently between three and four, regardless of the setting. The standard deviation for the serial interval distribution varied much more for the MLE method depending on the location. Garske et al. estimates indicate that the value was consistently smaller in the communities than in the ships. It is not clear exactly how to interpret this result. Further, we consistently see a large initial value for the reproductive number. In the ships, this value is higher and rapidly drops off, perhaps due to the close quarters and extremely rapid transmission that could take place in these very vulnerable populations. In the communities, the reproductive number tended to drop off later, typically around day thirty. This could be due to a larger initial susceptible population and more complicated dynamics for the disease to spread, leaving large pockets of susceptible individuals unexposed for a longer period of time than in the ships.

These results confirm the high pathogenicity of influenza and its ability to rapidly spread through populations. It also appears that the greatest difference between the spread of influenza in a closed population without the ability to implement control measures is a large initial reproductive number that declines rapidly. In more diffuse communities with complicated dynamics, it is likely that the reproductive number will not decline as rapidly.
